# A Preliminary Mixed-Method Investigation of Trust and Hidden Signals in Medical Consultations

**DOI:** 10.1371/journal.pone.0090941

**Published:** 2014-03-11

**Authors:** Silvia Riva, Marco Monti, Paola Iannello, Gabriella Pravettoni, Peter J. Schulz, Alessandro Antonietti

**Affiliations:** 1 Department of Health Sciences, Università degli Studi di Milano, Milan, Italy; 2 Institute of Communication and Health, Università della Svizzera Italiana, Lugano, Switzerland; 3 IBM Italia, Milan, Italy; 4 Università Vita e Salute San Raffaele, Milan, Italy; 5 Department for Adaptive Behaviour and Cognition, Max Planck Institute for Human Development, Berlin, Germany; 6 Department of Psychology, Università Cattolica del S. Cuore, Milan, Italy; 7 Psycho-Oncology Unit, Istituto Europeo Oncologico (IEO), Milan, Italy; University of Pittsburgh Medical Center, United States of America

## Abstract

**Background:**

Several factors influence patients' trust, and trust influences the doctor-patient relationship. Recent literature has investigated the quality of the personal relationship and its dynamics by considering the role of communication and the elements that influence trust giving in the frame of general practitioner (GP) consultations.

**Objective:**

We analysed certain aspects of the interaction between patients and GPs to understand trust formation and maintenance by focusing on communication channels. The impact of socio-demographic variables in trust relationships was also evaluated.

**Method:**

A cross-sectional design using concurrent mixed qualitative and quantitative research methods was employed. One hundred adults were involved in a semi-structured interview composed of both qualitative and quantitative items for descriptive and exploratory purposes. The study was conducted in six community-based departments adjacent to primary care clinics in Trento, Italy.

**Results:**

The findings revealed that patients trusted their GP to a high extent by relying on simple signals that were based on the quality of the one-to-one communication and on behavioural and relational patterns. Patients inferred the ability of their GP by adopting simple heuristics based mainly on the so-called social “honest signals” rather than on content-dependent features. Furthermore, socio-demographic variables affected trust: less literate and elderly people tended to trust more.

**Conclusions:**

This study is unique in attempting to explore the role of simple signals in trust relationships within medical consultation: people shape trust and give meaning to their relationships through a powerful channel of communication that orbits not around words but around social relations. The findings have implications for both clinicians and researchers. For doctors, these results suggest a way of thinking about encounters with patients. For researchers, the findings underline the importance of analysing some new key factors around trust for future investigations in medical practice and education.

## Introduction

### The Trust Relationship

The bond of trust between the patient and physician has been conceived as the essence of the diagnostic and therapeutic process [1]–[5]. A great deal of research has been devoted to analysing the role of trust in medical decision making and treatment choices [6]–[9]. Trust mediates positive outcomes including adherence to treatment and satisfaction [8], [10]–[11]. Patients' trust may help physicians make accurate diagnoses to provide optimal treatment [5], [7]. Trust also correlates positively with the acceptance of new medications, intentions to follow physicians' advice, perceived effectiveness of care, and improvements in self-reported health status [12]–[14].

Factors influencing the trust between the patient and the doctor include socio-demographic variables such as the patient's age, gender, health, and education, as well as the patient's attitude, behaviour, and delegation [13]–[17]. However, recent studies on general practitioner (GP)-patient relationships demonstrated the primacy of communication in clinical encounters to build trust over time [18]. Communication based on trust occurs throughout consultations—for example, in acts of deep inquiry or in acts of listening and response: when the doctor takes, cedes, or shares control or facilitates the conversation with patients; when the doctor adjusts shared information based on the level of trust to meet the needs of the patient; when emotions and preferences are integrated into a collaborative relationship. When physicians spend time educating, communicating with, and orienting patients, the patient's health outcomes improve [18]–[19].

### The Role of Social and Honest Signals

According with the current mainstream literature on patient-doctor communication, the quality of care that a patient receives is partially influenced by the physician's communication skills [20]–[21]. Doctors who are explanatory, show support and respect for the patient, and enable the patient's participation in care generally have patients who are more satisfied, are more compliant to treatment regimens, and experience better health following the consultation [20]. In addition, doctors' expressions of positive affect consistently predict more positive communication with their patients and better judgments (patients report being satisfied with care) [22]–[24].

In the frame of the current literature about patient-doctor relationships, an innovative approach reappraises the role of communication channels [25]–[26]. When people interact, they communicate through two main channels: verbal language and the “unspoken messages.” To be effective, the first channel requires that all of the actors involved in the interaction agree on the terms, contents, meanings, and expressions being used. This may be not the case when people are not experts in a specific domain (e.g., medical content). The second channel revolves not around words but around social cues. People may, even unconsciously, rely more on this second channel of communication, namely, on the non-linguistic, “social sense” [25] in relation to the complexity, domain specificity, and knowledge of the content that people have to handle [27].

Despite the fact that the current literature in patient-doctor communication is flourishing and proliferating, little research has investigated the interaction between verbal and non-verbal channels in patient-doctor communication. Many types of human behaviours can be reliably predicted from biologically based honest signals. These primordial primate signalling mechanisms—such as the amount of synchrony, mimicry, activity, and emphasis in communication—form an unconscious channel of communication among people. As Pentland claimed, “these social signals are not just a back channel or complement to our conscious language; they form a separate communication network that powerfully influences behaviour” [25]. Indeed, these honest signals have repercussions on our plans, goals, and values. By examining this primordial channel of communication, people can precisely predict outcomes of certain events, negotiate, make decisions, make agreements, and create stable relationships based on trust.

People are familiar with many types of human signals, according to Pentland: “Smiles, frowns, fast cars, and fancy clothes are all signals of who people are. In fact, this sort of signalling is most likely the basis of customs and ‘current culture’” [26]. People are sometimes conscious of these types of signals and often carefully decide to include them in communication. Because these signals are so frequently planned, people cannot rely on them because they may fail to be honest. As Pentland highlighted “People need to find signals that are processed unconsciously, or that are uncontrollable, before they can count them as honest and trustworthy. Once these signals are elaborated as honest and safe, people tend to trust and to generate process of delegation, especially when they address difficult matter or issues” [26]. Then, they become “honest signals.”

People can find several examples of honest signals. The main four categories of these signals, identified by the author, are:

- Influence. This concerns the impacts that each person produces on another in a social interaction. According to Pentland, “influence is measured by the extent to which people cause other persons' pattern of speaking to match their own pattern” [26].
- Mimicry. This refers to the “reflexive copying” [26] of one person by another within a conversation, resulting in an automatic exchange of agreement signals, smiles, exclamations, and head nodding during a conversation or in following someone with one's own eyes.
- Activity. This indicates a level of interest and excitement. In the medical context, attention to the problem, participative behaviour, and interest can represent examples of activity.
- Consistency. This is a signal of mental focus that refers to the capacity to comprehend emotions and feelings and to react adequately.

Each of these signals has its structures in the organisation of the brain and in human physiology. This may be why they are such reliable signals of our behavioural tendencies. By evaluating the precision and reliability of responses among people, the influence measure provides an evaluation of attentional mechanisms.

According to the Adaptive Behaviour and Cognition research group led by Gerd Gigerenzer, relying on what we called “honest signals” can be considered the outcome of an ecological strategy or heuristic that allows people to make decisions by exploiting the relevant information from their environment [28].

These honest signals influence critical activities such as negotiation, group decision making, and building trust relationships. Influence, mimicry, activity, and consistency play important roles in shaping social interactions and have evident repercussions on trust. In this perspective, the patient-doctor relationship represents an interesting scenario for research.

Because several factors can influence patients' trust, and trust can influence the doctor-patient relationships, complete models and theories about the role of trust and how it can be maintained and reinforced are needed [29]. In accordance with this point, current studies should evaluate the role of communication channels, which seem to represent a core and quite innovative aspect in the dynamics between patient and doctor to build a positive and trustful relationship.

### Objective

The aim was to analyse some aspects of the interaction between patients and GPs to understand trust formation and maintenance by focusing on communication channels. The impact of socio-demographic variables in trust relationships was also evaluated.

## Methods

A cross-sectional design using concurrent mixed qualitative and quantitative research methods was employed. We involved participants in semi-structured interviews composed of both qualitative and structured items for descriptive and exploratory purposes. The same respondents participated in both the quantitative and qualitative phases of the study. As noted by Creswell et al., a concurrent design is a powerful study design in which “the researcher seeks to compare both forms of data—quantitative and qualitative—to search for congruent and comparable findings” [30].

This research was conducted as part of a larger study into the meaning of “personal self-care” in the Autonomous Province of Trento (Northern Italy). The participants were recruited after the project received the Institutional Review Board (IRB)'s approvals from the Italian Primary Care Trust (PCT) of the Province of Trento. Each respondent provided written informed consent prior to participation. Parts of the results of the larger study that do not overlap this article have been published [31]–[32].

We entrusted the management of data storage to the *Department for Adaptive Behaviour and Cognition* at the Max Planck Institute for Human Development in Berlin, who supported the project with the use of the data collection and storing software.

### Participants

One hundred participants were purposively sampled from the six main local PCT departments of Trento Province and varied in terms of locality, gender, and age. As Onwuegbuzie reported [33], in most of the mixed methods studies with a concurrent design and identical samples, a convenience sample approximately 80–100 subjects is used [34]–[36]. The intention was to interview participants in a naturalistic and familiar environment. Typically, such an approach involves conducting individual interviews with a small number of respondents to explore their perspectives on a particular idea or situation and 100 participants represent a large sample in this type of research [33].

We used a convenience sample. In pilot studies, a convenience sample is typically used because it allows the researchers to obtain basic data and trends without the complications of using a randomised sample. We adopted a sample frame information with rigor and documented each stage of the sampling process. Even in the frame of a mixed design, we structured our research according to the purpose of sampling in qualitative research, which is not to establish a random or representative sample drawn from a population but to identify specific groups of people who either possess characteristics or live in circumstances relevant to the social phenomenon being studied [33]. The participants are identified because they enable exploration of a particular aspect of behaviour relevant to the research. This allows researchers to include a wide range of types of informants and also to select key informants with access to important sources of knowledge.

Furthermore, the structure of our sample can be considered sufficiently heterogeneous and representative according to the following conditions:

The epidemiological context of the Trento region is similar to that of other Italian regions;
The health system procedures applied in the Trento region are the same as in the other Italian regions. Under certain conditions, patients are able to choose which GP to register with—that is, according to their residence and to the availability of doctors who can accept them because the system has a restricted access and each doctor has a fixed number of patients under his or her department;
The sample is heterogeneous by gender, age, and the level of education to the same extent as in the other Italian regions;
The health operators of the Trento region have the same qualifications as in the other Italian regions.

We have no reason to suspect that possible biases affected the sampling procedure.

Recruitments were structured via waiting room leaflets, small posters, and direct contacts with patients. The researchers contacted patients who came to the local PCT department in the same period of the year by asking them to volunteer in the study by taking part in an interview. About half of the patients were recruited in the morning and half in the afternoon, so possible differences in job and family activities (which might lead people to prefer selectively a part of the day to come to the department because of his or her lack of duties at that time) should be excluded. Patients were recruited in land services adjacent to the departments. Finally, we used the same criteria to select cases and we excluded self-referral cases.

### Interview Design

A map of the interview is reported in Table 1. The sections of the interview are described in detail as follows.

**Table 1:** Map of the interview.

N°	Main Areas	Topic	Question	Type of questions
	SOCIO-DEMOGRAPHIC			
1		Age	How old are you?	Closed
2		Gender	(a) Female or (b) Male	Closed
3		Level of education	Which is your level of education? (a) Primary school, (b) Junior high school, (c) High school, (d) University	Closed
4		Job status	(a) Employed, (b) in retirement/Looking after family	Closed
5		Longstanding illness	Do you have any long-standing impairment, illness or disability?	Closed
	TRUST			
6		Long-term relationship	Do you have a long-lasting and trustful relationship with your GP? *(Add any additional comment)*	Closed
7		Trust in GP	How much trust do you have in your GP? *(Add any additional comment)*	Closed
8		Trust in Medicine	How much do you trust medicine? *(Add any additional comment)*	Closed
9		Trust in Health System	How much do you trust health system? *(Add any additional comment)*	Closed
10		Representation of Trust	Word associations	Word association
	SOCIAL SIGNALS			
11		Activity	Generally, your GP understand well and careful your health problems? *(Add any additional comment)*	Closed
12		Mimicry	My GP takes care about my fears (eg. fear for a new treatment to follow, fear for a surgery, fear for a new potential diagnosis) *(Add any additional comment)*	Closed
13		Consistency	My GP gives me a clear picture of my health status (eg., use of a transparent voice, understandable explanations) *(Add any additional comment)*	Closed
14		Influence	Do you usually argue about your treatment options with your GP?	Closed

Section 1—Demographic characteristics. The interview started with a series of questions pertaining to the respondent's age, gender, health status, occupation, education, and the presence of long-standing impairment, illness, or disability.

Section 2—Trust. This section is constituted by two parts. First, four questions were aimed at assessing the patient's level of trust. In particular, we articulated this aspect by asking the following questions:

Do you have a long-lasting and trustful relationship with your GP?
How much do you trust medicine?
How much do you trust the health care system?
How much do you trust your GP?

Answers were given on a 10-point scale from “not at all” to “completely.” The level of trust was measured according to the scores given by participants.

**Figure 1:**
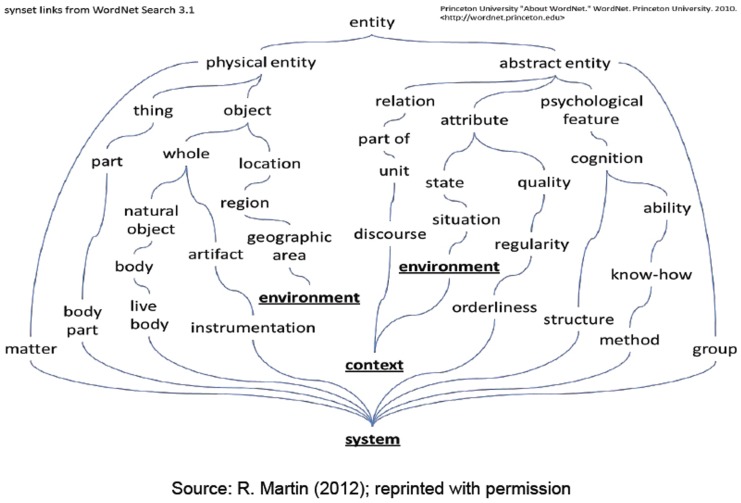
The IBM ICA Studio. Text mining and Natural Language Processing software usually integrate lexical resources like WordNet, a lexical database for the English language, developed starting from 1985 by the Princeton University, department of Psychology, under the direction of George A. Miller. WordNet groups nouns, verbs, adjectives and adverbs into sets of cognitive synonyms, each expressing a distinct concept. The concepts are interlinked by means of conceptual-semantic and lexical relationships.

Then, participants were invited to list five associations that usually came to mind when they thought about trust in a medical context. Answers were collected and lemmatised (e.g., drugs→drug), aggregated semantically (e.g., physicians, general practitioners, doctors→“doctor”; mistake, error→“mistake”) and were classified and processed by a text mining and natural language processing software (*ICA Studio*) [37]. The adopted analysis technique was the “continued associations method,” which has been shown by Szalay and Deese [38] to be a sensitive indicator of the meanings associated with people's mental representations for a wide variety of concepts. Thanks to the adopted software, we succeeded in extracting valuable information from the texts and, in particular, in identifying concepts represented by synsets, namely, groups of lemmas that are considered semantically equivalent. Synsets are linked to each other by specific semantic relationships (links) that can be represented in a hierarchical structure. In this way, each concept is enriched with the characteristics and meaning of nearby concepts according to the chosen ontological representation. Fig. 1 illustrates the synset links representation in Wordnet.

Section 3: Honest signals. Participants were asked about their experiences with their GP. According to Pentland's perspective, there are patterns in human behaviour that can reveal important aspects of a person's thoughts, such as people's intentions, opinions, and values. Observing behaviour and understanding these patterns can predict the outcomes of social situations, such as the type of interaction between a patient and GP and the value of trust in this relationship.

Participants were asked to endorse four statements linked to each social signal category:

Activity: “Generally, my GP interprets my health problems well and carefully.”

Mimicry: “My GP cares about my fears (e.g., fear for a new treatment to follow, fear of a surgery, fear of a new potential diagnosis).”

Consistency: “My GP gives me a clear picture of my health status (e.g., use of a transparent voice, understandable explanations).”

Influence: “I usually argue about my treatment options with my GP.”

Closed questions asking patients to rate their agreement toward a given sentence or to express an evaluation were based on a 10-point scale from “not at all” to “completely,” as reported in Table 2.

**Table 2:** Likert scale.

Not at all		Mostly not		Somewhat		Mostly yes		Completely	
1	2	3	4	5	6	7	8	9	10

The answers to closed questions might be enhanced by personal comments about individual experiences or opinions.

### Administration of the Interview

The interviews were conducted by three social scientists trained in qualitative research (two of whom are authors of this article: SR and MM). The respondents signed an informed consent to declare their agreement to take part in the study.

To facilitate the data collection and the subsequent analysis, the interviews were audio-registered and transcribed using Unipark [39], an online survey software for empirical research. This allowed us to track the responses and check for possible interactions and misunderstandings and to better organise the file of the answers using different formats (e.g., Excel data, SPSS data). Specifically, this software provided support to store participants' data, to manage the participants' access, to monitor the incoming results, and to request a report.

## Results

### Socio-Demographic Characteristics

Regarding the characteristics of our participants, the sample was rather equilibrated according to gender and employment status. The sample included 57 (95%; CI = 47.3–66.7) men and 43 (95%; CI = 33.3–52.7) women. Forty-five subjects (95%; CI = 46.58–66.02) were employed, and 45 (95%; CI = 35.25–54.75) were retired. Participants without longstanding illnesses were the majority (N = 67; 95%; CI = 57.78–76.22), and there were 33 (95%; CI = 23.78–42.22) participants with longstanding illnesses. The mean age of the participants was 52.7 yrs. (range = 24–88; SD = 15.4). We grouped participants into four age groups: “young” (N = 17, 95%; CI = 8–22), “young adults” (N = 20; 95%; CI = 22.12–40.28), “adults” (N = 28; 95%; CI = 19.92–37.68), and “seniors” (N = 25; 95%; CI = 16.51–33.49). Except for the youngest age group, the numbers of participants in the other age groups were similar. We also divided participants into four levels of schooling from “primary school” to “university degree.” Nearly 60% of participants had received a high-school diploma. The minority of participants (N = 13; 95%; CI = 5.02–17.38) had received a primary school certificate, and the other two groups (junior high school and degree) were quite homogeneous.

### Trust

**Figure 2:**
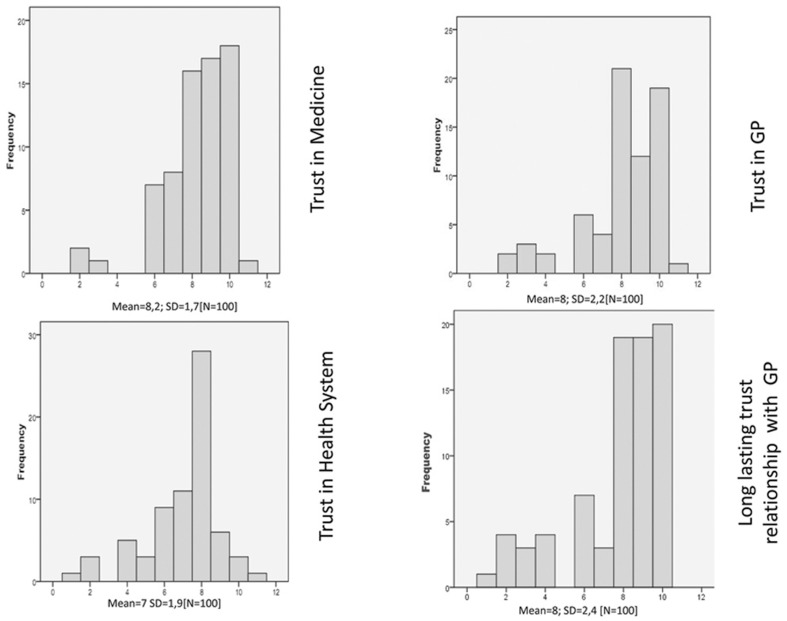
Trust in medical context.

The participants showed a high level of trust in medicine and in their own GP, reporting highly trustful interactions and a long-lasting relationship with ratings of 8 or 9 on the 10-point scale, where 8 and 9 represented a very high degree of trustful interaction (see Fig. 2). The distribution of answers concerning the national health system was in line with the Italian Annual National Survey on Health [40]. About half of respondents were not very satisfied in the health system (N = 45; 95%; CI 46.27–65.73), but the other respondents (N = 55, 95%; CI 45.25–64.75) considered the support from the national health system to be good or very good. The participants showed trust particularly in people and information with whom/which they were socially close. By collecting additional comments, it emerged that the GP represents a figure close to the direct experience and problems of people. The word “medicine” was conceived of as related to research and science and as representing an “engaging content” [41] that stimulated the interest of people by being a frequent topic of discussion in social media contexts and networks. On the other hand, the expression “national health system” represented something more disconnected and far from individuals' daily lives and direct experience.

### What Does Influence the Conferring of Trust?

The participants were asked to list five features that they considered central to trusting their GP. They were then asked to rank these features in order of importance.

We clustered the contents using *Sensigrafo*. Four main synsets were identified. The synset “Competence” referred to the aspects related to the GP's knowledge, expertise, and ability in making diagnoses and providing treatment. The synset “Communication and Relationships,” in the frame of the honest signals theory, referred to aspects including influence, mimicry, activity, or consistency (for example, the capacity of the GP to have empathy, to show interest in the patients' health problems, to take care of their worries, to show clarity of argumentations, to maintain a calm and patient approach, and to use an informal and friendly style). In this area, aspects concerning verbal and non-verbal communication were included. The synset “Accessibility” was connected with the timeliness of receiving treatment, good management of appointments, and punctuality. Finally, the synset “Health System” referred to the infrastructures, including facilitation for patients (e.g., transport services), educational activities (e.g., training for citizens), and level of innovation and performance of the system (e.g., territorial network collaboration with other hospitals and structures). Table 3 reports the percentage of answers dealing with competence-based aspects versus relationship- and communication-based features.

**Table 3:** Percentage of respondents' ranking of features affecting trust.

Synset	Rank Order of Features			Ranking Mean
	First	Second	Third	
Competence	25%	31%	26%	27.4%
Relationship and communication	51%	47%	51%	49.7%
Accessibility	24%	14%	14%	17.3%
Health system	0%	8%	9%	5.6%

**Figure 3:**
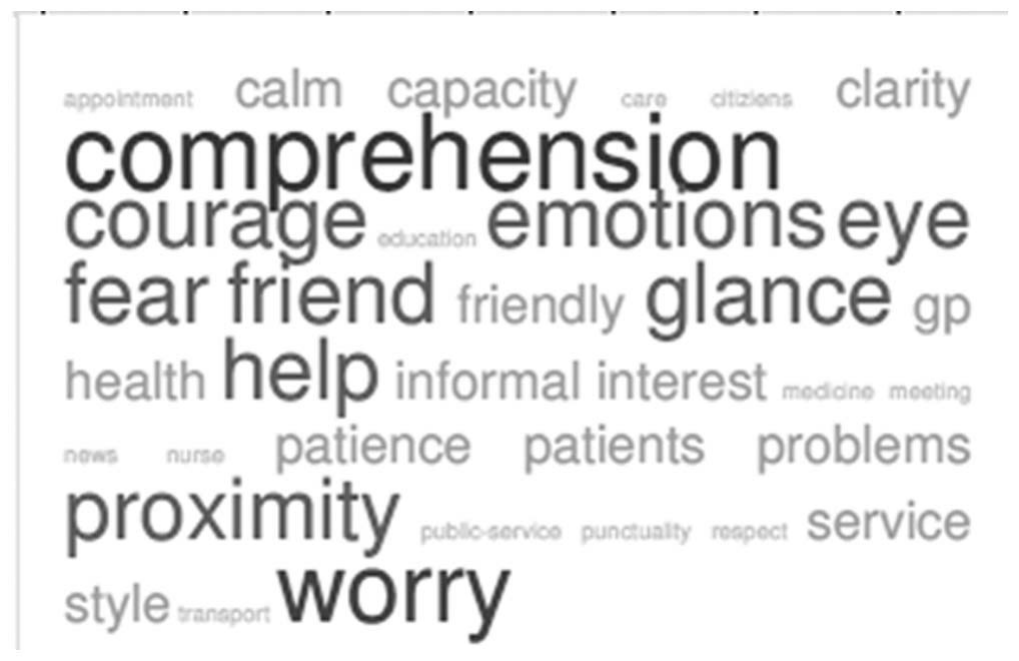
Tag Crowd about trusting own GP visualized by frequency.

In Table 3 features dealing with the quality of the patient-GP interaction (e.g., relationship and communication) were mentioned most frequently (N = 78; 95%; CI = 69.88–86.12) and were considered more important than features related to the GP's competence and expertise. The participants frequently stressed the ability of the GP to show verbal and non-verbal signals of comprehension and reciprocity. Aspects belonging to influence, mimicry, activity, and consistency were more frequently reported by the respondents and were considered to be core elements of maintaining a trustful relationship with their own GP. In particular, the following aspects emerged as very important:

1. comprehension of the patients' point of view—Activity (N = 42; 95%; CI = 32.33–51.67);
2. shared glance—Mimicry (N = 20; 95%; CI = 12.16–27.84);
3. understanding emotions of fear and reacting appropriately—Consistency (N = 15; 95%; CI = 8–22);
4. expressing words of encouragement—Activity (N = 15; 95%; CI = 8–22); and
5. reassuring and using an informal and friendly style —Influence (N = 8; 95%; CI = 2.68–13.32) (see Fig. 3).

### Social Honest Signals

In the third section of the interview, the participants were asked to evaluate some features of their relationships with their GPs. Particularly, they were asked whether their GP takes care of their fears, carefully evaluates their medical cases, shows interest in and knowledge about their health conditions, and/or whether patients argue with their GP about treatment decisions or whether the influence of the GP's opinion is more authoritative. We considered the patients' answers valid indicators of the presence of social signals that usually emerge in various aspects of verbal and non-verbal interactions: we called these signals “honest signals for the patients.”

The patients' representation of the GP relationship was very positive. The majority of participants considered their doctor careful (N = 78; 95%; CI = 69.88–86.12) and empathic, good at understanding emotions and feelings (N = 85; 95%; CI = 78–92). The participants reported high satisfaction, with ratings of 8 or 9 on the 10-step scale. Most of the respondents reported that they receive clear and understandable explanations from their GP; thus, most likely for this reason, they do not frequently argue with their GP's opinions and decisions (on average, more than half of the respondents do not discuss what their GP proposes to them).

### Correlations with Trust

“Social honest signal items” showed some positive correlations with the level of trust in the GP. A multiple linear regression was performed with all of the identified variables, as shown in Table 4.

**Table 4:** Coefficients of multiple linear regression analysis on trust levels.

Model	Standardised Regression	t	p	95% Confidence interval	
	Coefficient				
	Beta			Lower	Upper
(Costant)		−2,069	0,042	−3,45	−0,062
My GP takes care about my fears [Mimicry ]	0,223	3,421	0,001	0,09	0,342
Generally, your GP understand well and careful your health problems? [Activity	0,352	4,194	0,000	0,182	0,512
My GP gives me a clear picture of my health status [Consistency]	0,286	3,722	0,000	0,15	0,497
Do you usually argue about your treatment options with your GP? [Influence]	−0,013	−0,820	0,415	−0,45	0,19
Employment Status	0,1	1,056	0,295	−0,054	0,174
Sex	0,036	0,639	0,525	−0,046	0,089
Age	0,049	0,508	0,613	−0,042	0,071
Education	0,105	1,598	0,115	−0,009	0,081
Long standing illness	0,111	1,948	0,056	−0,002	0,147

Dependent variable: Trust: How much do you trust your GP?

The demographic variables (gender, age, education, and employment status) were not correlated with the level of trust and failed to emerge as independent predictors of trust. Understanding the patients' health problems well and carefully was the strongest predictor of trust (t = 4.19; p<.001). Clear explanations (t = 3.72; p<.001) and empathy in recognising emotions of fear (t = 3.42; p<.001) seemed to be independent predictors of trust.

Path analysis is an extension of multiple regression that goes beyond prediction and examines the relationships between independent variables to identify the direct and the indirect effects that they have on the dependent variable within a non-experimental design. As reported by the literature, a path analysis can be applied within the context of quantitative and mixed methods using a sample size with at least 80–100 subjects, depending on the number of free parameters/variables [42]. Having identified satisfaction, good care experiences, and continuity as key predictors, the primary concern was to identify the strength of their influence on trust and to determine a potential additional role of delegation.

**Figure 4:**
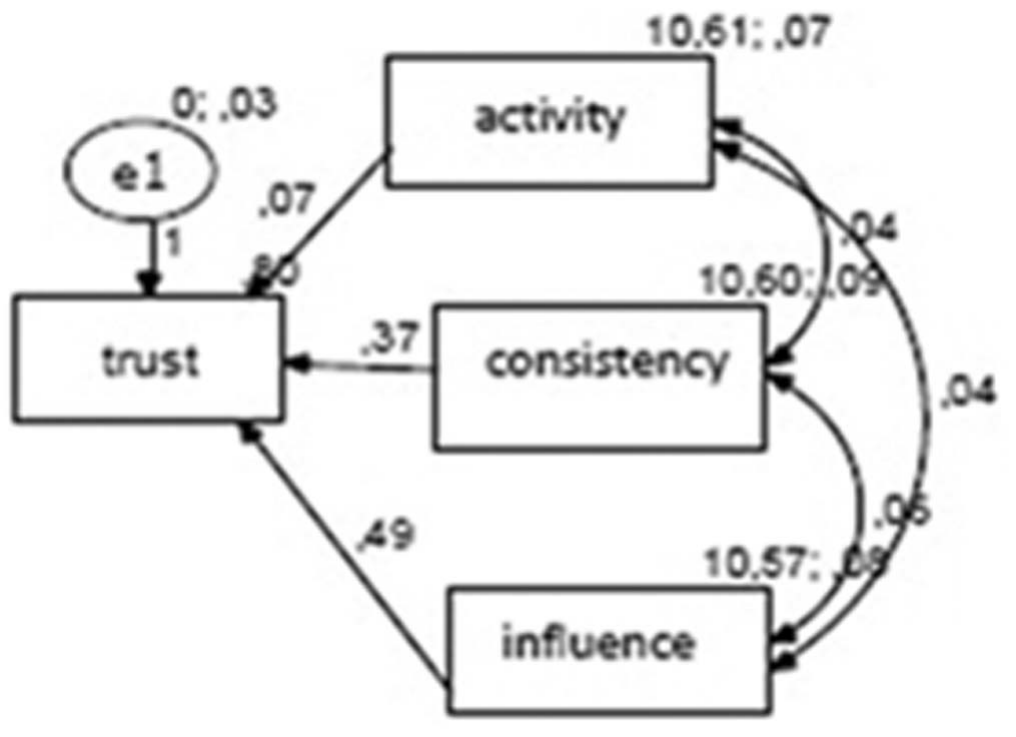
Output of the best-fitting path model.

The AMOS software (version 18) was used to construct an input path diagram representing the casual model and linking the variables of trust, good care, continuity, and satisfaction. The data were entered for 100 cases. Standardised beta coefficients were generated for all of the paths, and R^2^ values were generated for all of the endogenous variables. After having tested several models, the model that was the best for explaining the relationships among variables is reported in Fig. 4. The goodness of fit (CMIN, see the appendix) was x^2^ = 4.29, df = 3, p = .232. The p value is not significant and indicates a good fit. The NFI was .984, and the CFI was .999. The RMSEA value was .07, and it signifies a reasonable fit. In the parameter summary, we found that the variance was never negative among the variables and was always significant. The regression weights did not present a value of variance <.30. These results indicated that careful understanding is the best predictor of trust in this model. Good understanding, clear explanations, and empathy were inter-correlated variables.

### The Impact of Demographic Characteristics

We investigated the role of the demographic variables on trust and delegation. Regarding trust, we hypothesised that the less literate and competent respondents should be more likely to trust the GP to avoid mistakes and that the respondents with high-schooling and who were more knowledgeable about the subject should more likely trust their GPs to save time and effort. We grouped our participants into four age groups (young, young-adults, adults, and seniors) and into four levels of education (from primary school to university) as described in the methods section. Our data confirmed that the less literate people rely on their GP more than do literate people. Almost all of the respondents (12 out of the 13 subjects: 95%; CI = 77.25–106.75) in Group 1 (very low level of schooling) showed a high level of trust, and only 9 out of the 20 participants (95%; CI = −3.54–21.54) in Group 4 (very high level of schooling) reported a high level of trust. The association between trust and schooling was significant (X^2^ = 4.27, p<.005). Group 2 (junior high school) and 3 (high school) showed high levels of trust. No significant difference was found in relation to age and gender.

We also analysed the influence of age and gender on trust level. With respect to age, while Groups 2 (36–49 yrs., N = 30) and 3 (50–64 yrs., N = 28) showed similar distributions, major differences emerged in Groups 1 (22–35 yrs., N = 17) and 4 (over 65 yrs., N = 25). More than half of the young people (N = 12; 95%; CI = 43.48–88.52) and the great majority of the older people (N = 21; 95%; CI = 58.96–91.04) showed the highest level of trust. In relation to gender, we did not find particular differences in our distributions.

## Discussion

The objective of this study was to investigate the role of trust in the patient-doctor relationship. The results showed how trust and its constitutive elements are conceived by patients with a focus on communication signals. The study also highlighted the impact of demographic variables in a trusting relationship.

Even though this research does not claim absolute generalisations, we can describe some interesting findings in a context-bound sense that come from an active process of reflection given by the quantitative and qualitative data analysis.

As the literature has widely described, the results of this study indicate that trust comprises a main component in the relationship between the patient and doctor. The analyses showed that the patients have high levels of trust in their GP and medicine (conceived both as science and research), despite the fact that their trust in the health system in general is not so high. These data are in line with the results of the Italian survey on health [40] and with other recently published studies in the same context of our research [31]–[32].

Recently, various authors have argued that physicians' demonstration of care in their speech is related to greater satisfaction in the patient [21], more adherence to treatment [22], and better psychological adjustment to the illness [29]. The verbal behaviours that have been shown to be related to at least one positive patient outcome and that can be considered as caring are the following: expressed empathy, statements of reassurance and support, positive reinforcement, laughing and joking, courtesy, and psychosocial talk [21], [29].

The behaviours and attitudes described by physicians provide a repertoire of facilitators from which either person may draw to increase the likelihood that a trustful interaction will occur. As such, these findings suggest a more powerful and dynamic process based on “unspoken messages” [25]–[26] across verbal and non-verbal components. Simple and honest signals occur throughout interactions in medical consultations—for example, in acts of deep inquiry to understand patients' problems carefully; in listening and responding; when either person takes, cedes, or shares control or facilitates the other person's ability to do so; when physicians adjust the information to give patients a clear picture about their health status; and when emotions and fears are integrated into a collaboratively constructed decision. These signals were mentioned much more frequently and were considered more important than those related to the health system and even the competence and expertise of the GP. This means that participants adopt a simple ecological heuristic [28] and consider these former features to be valid cues for inferring the quality of the physicians' advice and for instilling trust. As recently described by Pentland [25], when people are aware that they are not especially well-equipped to judge and understand an unusual content (like that expressed in the technical medical language), they tend to rely on something that they know much better and that they consider closer to them: the “simple and honest signals.” Simple and honest signals are reliable indicators used by people to guide their own trust-generating attitude. This perspective suggests that social norms, awareness of others' reputations, and signals of trustworthiness from verbal and nonverbal communication influence decisions about trust, alongside the structural and dynamic aspects of the situations within which these individuals interact [43]. Aspects belonging to influence, mimicry, activity, and consistency were often mentioned by the respondents and were considered to be core elements for maintaining a trustful relationship with the GP.

Summarising this first part of the results, we observed that patients trust their GP greatly; they do so by adopting simple strategies (like heuristics), which are based on the quality of their one-to-one interaction and on behavioural and relational aspects. Patients infer the ability of their GP by relying much more on “honest signals” than on content-depending features.

We tried to estimate the weight of these honest signals through some specific questions pertaining to the patient-doctor relationship with a path analysis. Careful understanding, clear explanations, and empathy appeared to be independent predictors of trust. More precisely, the path analysis results are important because they describe the context of the patient-doctor consultation. In such a context, people ask experts questions to learn more about a topic (e.g., a diagnosis, a drug's use) because they do not have this specific knowledge. To rely on experts, verbal and nonverbal cues can offer a look into a person's likely trustworthiness. This has been known for years, but the cues that convey trustworthiness or untrustworthiness have remained a mystery. In the literature, we have some interesting works in some specific contexts (e.g., work in the music fields) [44], but the analysis of these cues is quite innovative in the medical context. By collecting data from the interviews, we realised that there is not one single non-verbal cue that determines a person's trustworthiness but that trustworthiness comprises a set of cues. There is no one golden cue. Rather, a coordination of cues—particularly cues related to carefulness, clearness, and empathy—is what matters.

Finally, we evaluated demographic differences in the trust relationship. In accordance with the recent literature [15]–[16], trust was found more greatly in less literate people in comparison with high-schooling participants and in the elderly population in comparison with younger participants.

### Limitations and Strengths of the Study

This is a preliminary and exploratory study, and the present findings require further investigation. The study has several limitations. First, the size of our sample, composed of 100 participants from clinics within the district of Trento, even though sufficient to carry out the statistical analyses we used, might be too small and localised to generalise to other settings. Second, our findings reflect the mutual influence between the physician and patient without examining how these communication signals started. For example, were patients more trusting because of the physicians' patient-centred communication or were physicians more supportive because the patients were asking questions, expressing concerns, stating preferences, and eliciting the interaction? Third, we acknowledge that doctor-patient trust and decisions outcomes can be affected by other variables not examined in this study including the patient's health status, longstanding relationships, the reason for the visit, and type of health care facility.

This study focused on the investigation of the communication's hidden dynamics rather than on the putative outcome. We tried our best to design interviews with questions that could reveal real-life communication dynamics. Nonetheless, we may expect different communication outcomes in real situations.

Last but not least, there were limitations in using the continued associations methodology; in particular, we noticed individual variations in people's ability to “think aloud” during the interview [45]: some individuals considered thinking aloud a relatively easy task but others experienced great difficulty. As a result, the conclusions made on the analysis of communication signals are formed with some caution.

In spite of these limitations, this study represents a first and unique attempt to explore the role of honest and simple signals in trust building within a medical consultation relationship, particularly, in investigating the role of doctors' attitudes and behaviours in facilitating patient communication.

The investigation reinforces the mainstream of current eminent literature in the field of communication studies in the health context: doctors' attitudes and behaviours exert considerable influence over the patients [20]–[22]. Where doctors are more patient-centred, patients are more involved and trustful. Particularly, in this study we showed that physicians reinforce patients' trust relationships when they are more informative, accommodating, and supportive using simple and honest signals. This is something new and innovative in the field. This study provides new insight on how to evaluate the patterns of unconscious social signals that form coherent and discrete channels of communication and have an impact on trust building. Pentland suggested that these social signals enable “social intelligence” as a powerful way to understand and read social networks, interpersonal relationships, and organisational contexts [25].

Second, doctors' expressions of honest signals consistently predicted that trust impacted more positively on patients' communication and judgments. These results likely reflect the dynamics of communicative reciprocity and mutual influence in medical encounters [20].

Fortunately, communication is a skill that can be taught. Doctors' communication with their patients can be improved with training to make them aware of the importance of simple and honest signals in medical encounters. For example, training medical students and doctors on verbal and non-verbal communication skills, body language, and other extra-linguistic cues in doctor-patient interactions may improve the quality of communication with patients from different backgrounds, and we expect that patients will benefit from these inclusions. The doctors' enhanced awareness of these communication features will permit them to best align their communication style to the patients' preferences and capabilities. Additional work in this area will encourage interventions that may enhance diversity among medical students to better address the extent to which a physician's own style affects the patient-physician communication and perceptions of care.

## Conclusions

The framework from this study might be helpful both to researchers and policymakers. For researchers, the study can lead them to identify the key factors that need to be considered for future research in this area and should substitute a piecemeal approach to this complex topic. Forthcoming studies should look in depth at various elements of communication such as the weight of honest signals in human interaction, consultation experiences, and communication channels.

For policymakers, this study can help to understand how—and under what circumstances—trust can be reinforced or undermined over time. Some changes of the organisation and delivery of PCT in Italy (but also in Europe) have the potential to decrease trust strikingly, as observed by national surveys (for example, because of the strict appointment management system, the limited resources for the outpatient treatment for the elderly population, or the lack of support in case of emergency). In these situations, patients are increasingly likely to be consulting unfamiliar health professionals. Although trust can be stimulated outside of ongoing GP-patient relationships, the aspects of clearness of information, attention to the problem, and empathy (all of which were found to promote patient trust) are more likely to be present when care is given by the same GP over time, which we also underlined in our study. This suggests that some of the current policies toward increased access and choice in primary care, at the expense of ongoing interpersonal continuity, may undermine patient trust and can damage the relationship between the patient and the doctor. Ways of solving this problem in primary care could include encouraging GPs to facilitate access to the patients and putting practice systems in place to ensure that this is made easy for the patient (for example, flexible appointment booking systems).

Nonetheless, this research provides a theoretical basis for previous descriptions of the development of the GP-patient trust relationship, and these findings can also generate a new set of understandings concerning the dynamics of trust in encounters and relationships.
